# Post-infectious inflammatory response syndrome (PIIRS): Dissociation of T-cell-macrophage signaling in previously healthy individuals with cryptococcal fungal meningoencephalitis

**DOI:** 10.14800/Macrophage.1078

**Published:** 2015-11-23

**Authors:** Peter R. Williamson

**Affiliations:** Laboratory of Clinical Infectious Diseases, National Institute of Allergy and Infectious Diseases, National Institutes of Health, Bethesda, MD 20892 USA

## Abstract

*Cryptococcus* is an important cause of central nervous system infections in both immunocompromised patients such as those with HIV/AIDS as well as previously healthy individuals. Deficiencies in T-cell activation are well-known to be highly associated with host susceptibility in HIV/AIDS as well in animal modeling studies, resulting in poor microbiological control and little host inflammation. However, recent studies conducted in human patients have demonstrated roles for macrophage signaling defects as an important association with disease susceptibility. For example, an autoantibody to granulocyte monocyte stimulating factor (GMCSF) resulted in defective STAT5 signaling and susceptibility to cryptococcosis. In addition, severe cases of cryptococcal meningo-encephalitis in previously healthy patients, with or without anti-GMCSF autoantibody, developed a highly activated intrathecal T-cell population but had defects in effective macrophage polarization. Intrathecal inflammation correlated with neurological damage, measured by the axonal damage protein, neurofilament light chain 1. Based on these studies, we propose a new syndrome of cryptococcal post-infectious inflammatory response syndrome (PIIRS) defined in previously healthy patients with cryptococcal meningo-encephalitis as the presence of a poor clinical response in the setting of at least 1 month of amphotericin-based fungicidal therapy and sterile cerebrospinal cultures. These findings are discussed in light of the potential for improving therapy.

*Cryptococcus neoformans* is an important cause of HIV-related disease worldwide with up to a half a million deaths globally ^[1]^. As highly active anti-retroviral therapy has become pervasive in developed countries such as the U.S., HIV-related disease as decreased by about half, although non-HIV related disease has remained persistent ^[2]^. Mouse modeling studies have provided extensive understanding of the role of mammalian immunity to the fungus. For example, the role of innate signaling of dendritic cells by toll-receptors TLR2 and TLR9 was established in mouse models for pulmonary control of the fungus ^[3, 4]^. In addition, CD4 and CD8 cells in adaptive immunity was established in mouse pulmonary models ^[5, 6]^ as well as the role of Th1 protective immunity in neurodissemination ^[7-9]^. More recently, the importance of the role of classically activated macrophages (M1) has been shown to be important in the control of *C. neoformans* infections with IL-4/IL-13 dependent alternatively activated (M2) macrophages associated with uncontrolled cerebral disease ^[10]^.

However, while essential and decisive for mechanistic modeling, mouse models have limitations. For example, different mouse strains have a highly variable range of immune responses to most infections. In regards to cryptococcal disease, mouse strains known to have a relative non-protective phenotype such as C57BL/6J have a greater Th2 bias than resistant strains and produce pulmonary neutrophilia and eosinophilia, which is not characteristic of human infections. In contrast, humans tend toward a histiocytic response with giant cell formation, depending on the degree of residual cellular immunity in the infected patient ^[11-13]^. This suggests a need to conduct immunological studies in the human host during natural infections to assess species-specific immune responses. Susceptibility to human cryptococcal infections is best known to be related to T-cell defects, mediated either by HIV/AIDS-mediated depletion or that due to immune suppression by agents such as calcineurin inhibitors in organ transplant recipients ^[14]^ or inflammatory disorders treated with corticosteroids. Genetic susceptibility has also been reported due to T-cell defects in Good's syndrome ^[15]^ or haploinsufficiency of the hematopoietic transcription factor GATA2 ^[16]^. Diseases associated with T-cell defects such as HIV have high fungal burdens due to defects in cellular immunity; and response rates have shown correlation with pathogen clearance from the cerebral spinal fluid (CSF) ^[17]^. Approaches have used fungicidal drugs ^[18]^ with the adjunctive Th1-polarizing cytokine interferon-γ (IFN-γ ^[19, 20]^. However, restoration of immune dysfunction in HIV-infected individuals after anti-retroviral therapy results in improved T-cell but can also produce a cryptococcal immune reconstitution syndrome (cIRIS), accompanied by increased macrophage activation that results in significant dysfunctional immune damage ^[21]^. Excessive inflammatory responses are particularly damaging within the spatial confines of the central nervous system, where cerebral edema mediated by inflammation can result in neurological damage and death from brain herniation ^[22]^.

In addition to immunosuppressed patients, central nervous system (CNS) cryptococcal disease occurs in a significant population of previously-healthy (non-HIV) individuals and has an estimated mortality 10-30% ^[23, 24]^. Similar to the experience in HIV patients, rates of microbiological clearance predict clinical outcome ^[25]^. However, the role of the immune system has not been examined in this population. This has led to conflicting approaches based on HIV paradigms, such as adjunctive IFN-γ ^[26]^. Alternatively steroids has been used to suppress inflammation in non-HIV patients with *C. gattii* infections ^[27]^.

However, data is increasingly showing a role for macrophage dysfunction in susceptibility to cryptococcal infections in these apparently immunocompetent individuals. Historically, pulmonary and meningeal cryptococcal disease has been associated with pulmonary alveolar proteinosis (PAP) ^[28-30]^, a pulmonary disease of poor secretion clearance by lung macrophages. PAP has recently been shown to be associated with autoantibodies to granulocyte-monocyte stimulating factor (anti-GMCSF) by reproducing the disease in macaques after inoculation of anti-GMCSF antibodies from human patients ^[31]^. GMCSF is an important M1-polarizing cytokine which results in macrophage phagocytic and pathogen cidal actions, in addition to pulmonary secretion clearance ^[32]^. Antibody to GMCSF in patients with cryptococcal meningitis results in defective signaling of STAT5 macrophage activation pathways ^[33]^. Anti-GMCSF autoantibodies appear to be particularly associated with infections with a closely related cryptococcal species, *C. gattii*, which has resulted in a multi-year outbreak in the pacific northwest of the US and Canada ^[34]^.

More recently, in a cohort of CNS cryptococcal disease in previously healthy individuals, T-cell inflammation led to increased levels of detectable neurofilament light chain-1, a biomarker of axonal damage ^[35]^. This leads us to call this syndrome Post-infectious Inflammatory Response Syndrome (PIIRS), defined by refractory disease constituted by continued poor or deteriorating mental status despite sterile CSF after 1 month of amphotericin-based fungicidal therapy. Interestingly, unlike other susceptible patient populations, the dendritic cell-T-cell synapse was intact, facilitating a robust IFN-γ response. Cellular and soluble markers from patients with refractory, severe CSF disease further suggested a potentially damaging immune response from T-cell activation with robust in situ expression of T-cell activation markers such as HLA-DR and soluble markers such as sCD27, IFN-γ and IL-6. However, regardless of the presence of anti-GMCSF antibody, an alternatively activated M2 macrophage phenotype was exhibited in brain tissue biopsies as well as in autopsies, demonstrated by expression of the M2 marker CD200R1 and defective expression of the M1 marker iNOS. Soluble markers of M2 activation such as IL-10 were also elevated but M1 cytokines TNF-α and IL-12 were not elevated, further suggesting an alternatively activated macrophage phenotype.

In conclusion, while investigations of intracellular pathogens such as *Cryptococcus* has traditionally implicated defective T-cell signaling in disease susceptibility, more recently, the presence of macrophage signaling defects could define new disease types and guide rational therapeutic strategies. Furthermore, while animal studies are an important guide to potential mechanisms of disease susceptibility, studies conducted in humans may more accurately model disease susceptibilities encountered ‘in real life’ patients and help guide therapy of difficult CNS infections such as cryptococcosis.

## Figures and Tables

**Figure 1:**
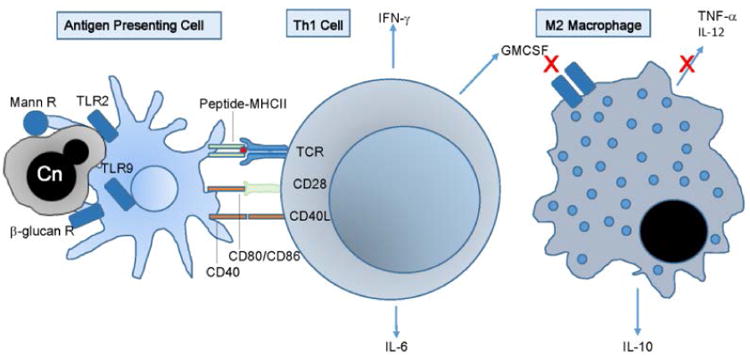
Model of immune signaling in previously healthy patients with a cryptococcal Post-infectious Inflammatory Response Syndrome (PIIRS), demonstrating activation of the antigen presenting cell-T-cell synapse but an alternatively activated M2 macrophage phenotype The encapsulated fungus and lysed fungal particles of *C. neoformans* (Cn) activates dendritic cells via the mannose receptor (Mann R), B-glucan receptor (b-glucan R) and Toll-like receptors 2 and 9 (TLR2, 9), resulting in T-cell activation with release of Th1-related cytokines such as IFN-γ and GMCSF and inflammatory IL-6. However, in this patient population, macrophages respond poorly, in some cases due to a GMCSF autoantibody blockade, resulting in IL-10 production but defective TNF-α and IL-12 secretion.
